# High prevalence of asymptomatic malaria infections: a cross-sectional study in rural areas in six departments in Haiti

**DOI:** 10.1186/s12936-015-1051-2

**Published:** 2015-12-21

**Authors:** Maha A. Elbadry, Basima Al-Khedery, Massimiliano S. Tagliamonte, Charles A. Yowell, Christian P. Raccurt, Alexandre Existe, Jacques Boncy, Thomas A. Weppelmann, Valery E. M. Beau De Rochars, Jean F. Lemoine, Bernard A. Okech, John B. Dame

**Affiliations:** Department of Environmental and Global Health, College of Public Health and Health Professions, University of Florida, Gainesville, USA; Emerging Pathogen Institute, University of Florida, Gainesville, FL USA; Interdisciplinary Center for Biotechnology Research (ICBR), University of Florida, Gainesville, USA; Department of Infectious Diseases and Pathology, College of Veterinary Medicine, University of Florida, Gainesville, USA; Laboratoire National de Santé Publique (LNSP), Ministère de la Santé Publique et de la Population (MSPP), Port-au-Prince, Haiti; Department of Health Services Research, Management and Policy, College of Public Health and Health Professions, University of Florida, Gainesville, USA; Programme National de Contrôle de la Malaria, Port-au-Prince, Haiti

**Keywords:** Malaria, Haiti, qRT-PCR, Diagnostic tools, *Plasmodium falciparum*, RDT

## Abstract

**Background:**

Public health measures are poised for transition from malaria control to malaria elimination on the island of Hispaniola. Assessment of the reservoir of asymptomatic infections from which acute malaria cases may derive is critical to plan and evaluate elimination efforts. Current field technology is ill suited for detecting sub-microscopic infections, thus highly sensitive survey methods capable of detecting virtually all infections are needed. In this study the prevalence of infection with *Plasmodium falciparum* was determined in patients seeking medical care primarily for non-febrile conditions in six departments in Haiti using a newly designed qRT-PCR-based assay.

**Methods:**

Three different methods of parasite detection were compared to assess their utility in approximating the prevalence of *P. falciparum* infections in the population: malaria rapid diagnostic test (RDT) designed to detect histidine-rich protein 2 (HRP2), thick smear microscopy, and a quantitative reverse transcription polymerase chain reaction (qRT-PCR) assay based upon the small sub-unit ribosomal RNA. The limit of detection of the qRT-PCR assay utilized was 0.0003 parasite/µL of blood. Venous blood was obtained from a total of 563 subjects from six departments in Haiti, all of whom were seeking medical attention without complaints consistent with malaria. Each subject was questioned for knowledge and behaviour using demographic and epidemiological survey to identify risk factors for disease transmission.

**Results:**

Among the 563 samples tested, ten and 16 were found positive for malaria by RDT and microscopy, respectively. Using the qRT-PCR test to assess the infection status of these subjects, an additional 92 were identified for a total of 108. Based upon the qRT-PCR assay results, a wide variation in prevalence of infection in asymptomatic subjects was seen between geographic locations ranging from 4–41 %. The prevalence of infection was highest in the Grand Anse, Nord and Sud-Est Departments, and demographic data from questionnaires provide evidence for focal disease transmission.

**Conclusions:**

The qRT-PCR assay is sufficiently sensitive to identify an unexpectedly large number of asymptomatic, submicroscopic infections. Identifying and clearing these infections presents a significant challenge to both control and elimination efforts, but the qRT-PCR assay offers a reliable method to identify them.

## Background

Hispaniola is the only Caribbean island where malaria is endemic, and the greatest burden is borne by Haiti, located on the western half of the island [1]. *Anopheles albimanus* is the predominant vector transmitting malaria on Hispaniola, and the causative agent is *Plasmodium falciparum* [2]. The national policy of the Haitian Ministry of Health [Ministère de la Santé Publique et de la Population (MSPP)] is to screen individuals with symptoms consistent with malaria using one of the WHO recommended rapid diagnostic tests (RDT); if positive, two slides each carrying thick and thin blood smear are obtained; one is read by local hospital technicians, while the second is sent to the national laboratory in Delmas [Laboratoire National de la Santé Publique (LNSP)] for confirmation. Although microscopy remains globally the gold standard for diagnosing malaria [3, 4], the accuracy of slide reading and their quality control remains questionable in Haiti [5]. Malaria remains high on the differential list of non-respiratory acute febrile illnesses in Haiti, despite the fact that in recent studies only about 3–17 % of these cases are confirmed as malaria by either microscopy or RDT [6, 7]. Official reporting of malaria cases to the LNSP is recognized as under-representing the true burden of malaria [2].

Since the vast majority of malaria cases on Hispaniola occur in Haiti, it should remain the focus of malaria control and future eradication efforts [2, 8, 9]. Eradication of malaria from Hispaniola is viewed as a reasonable goal, considering that it is an island isolated geographically from other malarious lands. Further, the disease is transmitted by a comparatively poor, exophilic vector [10], and chloroquine-sensitive *P. falciparum* appears to be the single agent of malaria [2, 9, 11]. However, the effort to eliminate malaria on Hispaniola will face frequent natural disasters, poor infrastructure, lack of skilled laboratory technicians, and geographic barriers presented by mountainous terrain, combined with limited resources available to people seeking medical help and their negative perception of the value of the healthcare provided to them [12]. All of these factors have hampered past efforts at control [2].

Rapid diagnostic tests have been identified as a key component of the current control strategy to prepare for eradication, thus keeping microscopy as only a confirmatory secondary test [13]. RDTs have a ≥75 % sensitivity at 200 parasites/µL of blood [14] with a false positive rate ≤10 % and an invalid rate ≤5 %, according to WHO published guidelines. In July 2010 Haiti MSPP approved the use of three RDTs in the country: First Response Malaria Ag HRP2 (Premier Medical Corporation Ltd., Watchung, NJ, USA), CareStart Malaria HRP2 (Pf) (Access Bio, Inc, Monmouth Junction, NJ, USA), and SD Bioline Malaria Ag Pf (Standard Diagnostics, Inc., Yongin, Korea) [15].

Moving beyond the malaria control phase to the elimination phase, it is suspected that in comparison to the whole population carrying malaria parasites, the febrile cases represent only the tip of the iceberg. The sub-population harbouring asymptomatic, sub-microscopic infections remains obscure. A recent review has described the potential for *P. falciparum* to persist in an asymptomatic host for more than a decade, making these infections potentially very important in elimination efforts [16]. Further, the importance of these asymptomatic infections rises when transmission levels are low such as in Haiti, where the overall official prevalence seems to range between 0.1–1 % [2, 17]. Despite the low prevalence of febrile cases, multiple studies have pointed out the high prevalence of asymptomatic carriers using only microscopy for diagnosis [7, 18]. Additional sub-microscopic infections remain unidentified, and will require implementing new survey tools capable of identifying them.

For the past five years malaria transmission in Haiti has been fairly stable, with a promising low level of overall transmission. However, in Africa and other areas around the world  where the transmission levels drop significantly, substantial numbers of sub-microscopic carriers remain, enabling the parasite to restart transmission when conditions are right [19–22]. In Tanzania, the prevalence of asymptomatic malaria infections as determined by microscopy was 1.9 %, whereas by real-time quantitative nucleic acid sequence-based amplification with a lower limit of detection, it was 32.5 % [22]. In this study a scheme is presented for testing field-collected samples from remote and undeveloped areas in Haiti to identify sub-microscopic infections using qRT-PCR in an attempt to further understand disease transmission and associated risk factors.

## Methods

### Blood samples

This study was conducted in accordance with institutional review board guidelines and requirements of the University of Florida and the ethical review board of the Haitian MSPP after obtaining all permits and approvals (UF IRB 201400202; UF IRB201400224; MSPP Ref. 1314-12; MSPP Ref. 1314-62). Subjects were recruited through a cross-sectional sampling of reproductive age women across Haiti in gynecology and maternity clinics. Study team collaborated with NGOs and government dispensaries to enrol women for free malaria screening. Each participant during the waiting time for enrolment was asked to answer a demographic survey in Haitian Creole. The questionnaire included general demographic and behaviour questions such as gender, age, geographic residency, history of travel, sleeping pattern (outdoor and/or indoor), presence of window screens, use of insecticide-treated bed nets, presence of bed nets whether treated or not, and history of malaria transmission for the participant and household members. Each questionnaire was labelled with a unique identifying number that matched the consent form and sample to provide de-identified data for analysis.

**Fig. 1:**
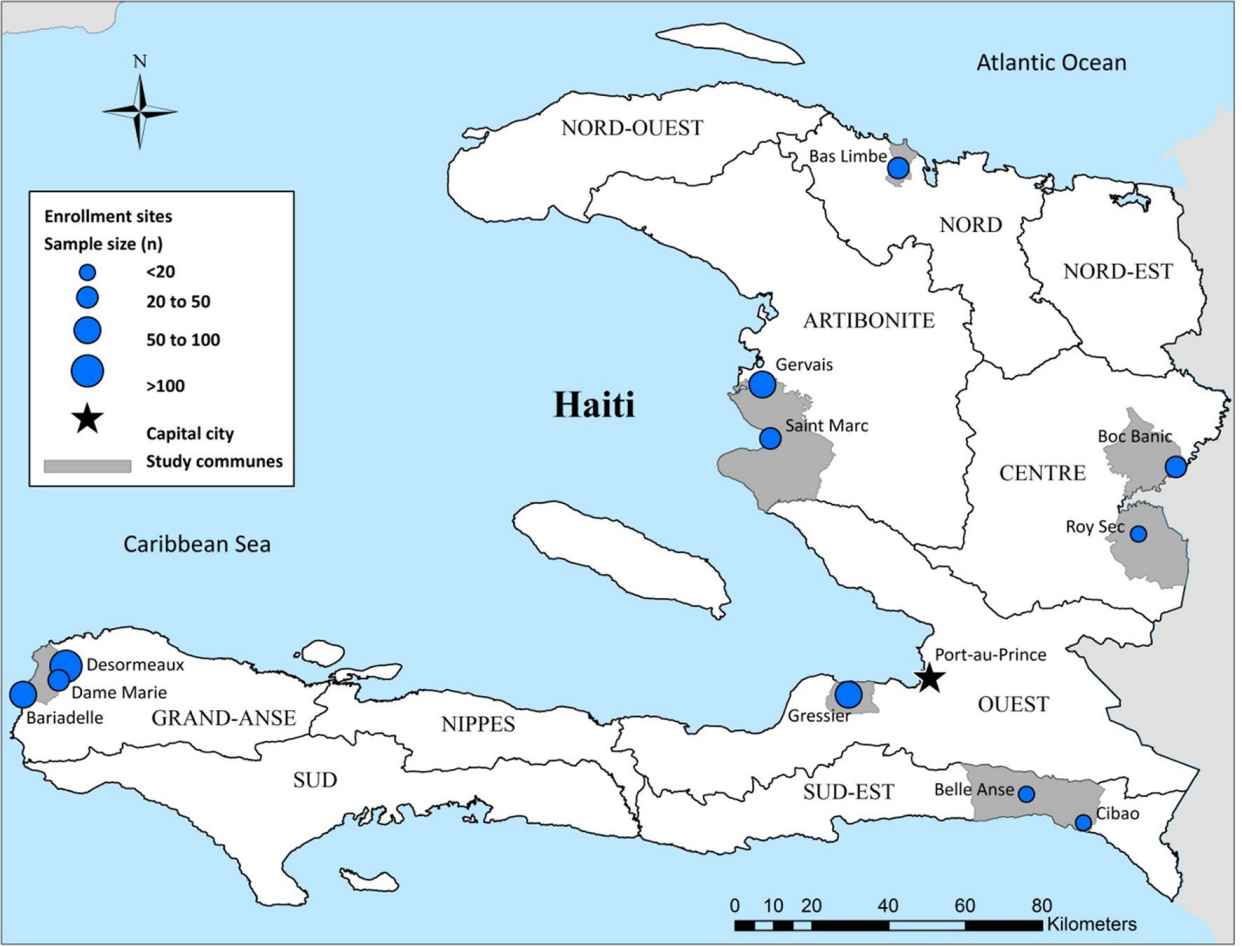
Geographic location of enrollment sites

Study sites shown in Fig. 1 were selected to cover diverse settings across the entire country of Haiti, including areas of high and low transmission. Subjects living in rural sites located in six of the ten departments, (Artibonite, Central Plateau, Grand Anse, Nord, Ouest, Sud-Est) were screened. Historically, malaria transmission has been reported in the Nord, Artibonite and Sud-Est Departments, and Grand Anse experienced an elevated level of transmission beginning in July 2014. In contrast, the Ouest and Central Plateau were known to be areas of low transmission. Between August 2014 and March 2015 a total of 563 venous blood samples were collected in an EDTA-coated, purple-top Vacutainer tube from attendants of maternity and gynecology clinics. Blood was mixed gently within the collection tube and 400 µL were transferred in a cryotube pre-filled with 1200 µL of DNA/RNA Shield™ (DRS, ZymoResearch, Inc.) for preservation of RNA and DNA. Approximately 25–50 µL of blood were used to make two microscopic slides (thick and thin smear) and run a CareStart Malaria HRP2 RDT. RDT results were recorded immediately in a study logbook. Microscopic slides were left to air-dry then Giemsa stained, following CDC protocol [23]. Whole blood in DRS was maintained at 4 °C or on wet ice continuously until placed at −20 °C upon return to the University of Florida laboratory in Gressier. The average travel time for the study team for sample collection, storage and sample freezing ranged from five to 15 days. Samples were shipped frozen on blue ice to the University of Florida, Gainesville, FL, USA.

### qRT-PCR assay design

A set of primers and a probe were designed to develop a highly sensitive molecular test to detect the presence of *P. falciparum* small sub-unit ribosomal RNA type A [24, 25]. These reagents were developed by aligning the small sub-unit rRNA gene sequences from the four most common *Plasmodium* spp. infecting humans (*Plasmodiumovale, P. falciparum, Plasmodium malariae, Plasmodiumvivax*) along with human small sub-unit rRNA using ClustalW2 software, and selecting specific primer pairs and probe using Integrated DNA Technologies, Inc. software. Care was taken to assure that the primers and probe would detect the small sub-unit rRNA, which is present predominantly in *P. falciparum* blood stage parasites. The following primers were selected for use in the qRT-PCR assay employed here: forward (5-GATACCGTCGTAATCTTAACCATAAAC-3), reverse (5-AAGGTACTGAAGGAAGCAATCT-3) and probe (6-FAM-ACACTTTCATCCAACACCTAGTCGGC-BHQ-1). The specificity of the primers was confirmed experimentally with RNA from *P. falciparum* clone 3D7 and RNA from uninfected human blood.

### Sample processing and qRT-PCR assay

Total RNA was extracted from 800 µL of the mixture of blood preserved in DRS using the Quick-RNA™ MiniPrep Kit following the RNA isolation from red blood cells protocol (Zymo Research) in a PCR workstation. The RNA was eluted in a volume of 50 µL of which 5 µL was used in the qRT-PCR reaction. Total nucleic acid extraction was also performed from blood samples in DRS using the same kit, but following a modification recommended by the manufacturer for obtaining total nucleic acids. Total nucleic acids were also eluted in 50 µL of which 5 µL was used in subsequent qPCR and qRT-PCR reactions.

The qRT-PCR was conducted using the Express One-Step SuperScript qRT-PCR Kit (Invitrogen) following manufacturer’s instructions with a final concentration of 0.4 µM of each primer and 0.2 µM for the probe in a 20 µL reaction. The PCR master mix preparation was conducted in a separate PCR work station. Amplification and real-time measurements were performed on a CFX96 Touch ™ Real-Time PCR Detection system (Bio-Rad). Reaction conditions were as follows: 20 min at 58 °C for reverse transcription step, one cycle of 2 min at 95 °C for Taq DNA Polymerase activation and 40 cycles of 95 °C for 15 s, 60 °C for 1 min for annealing and extension of amplified product. Every PCR run was conducted with two negative controls and one positive control. Samples were tested in separate batches where those with positive RDT results were extracted and tested separate from the ones with negative RDTs to reduce the possibility of cross contamination.

### *In vitro* limit of detection

A synchronous culture of *P. falciparum* 3D7 free of mature parasites and most culture debris was prepared as follows to obtain parasitized cells containing ring stage parasites, the developmental stage expected in the peripheral blood of infected patients. A fresh, semi-synchronous culture was prepared by the sorbitol method [26] and mature schizonts collected on a magnetic column [27]. The mature schizonts were released from the column and incubated with fresh red blood cells for 12–15 h to allow release of merozoites and re-invasion. The mature parasites that had not released merozoites were removed using a second magnetic column. The parasitaemia of the ring stage parasites in culture which passed through the column was determined using both microscopy and fluorescence activated cell sorting, and the number of parasites per volume of culture calculated based upon the number of red blood cells per volume of culture determined using a Coulter counter. Counted parasitized red cells from culture were mixed with whole human blood to prepare an initial sample containing 10^5^ parasites/ml.

A standard curve was prepared from RNA extracted from the initial sample containing 10^5^ parasites/ml and serially diluted in RNase/DNase free water in intervals of tenfold dilution with the final dilution equivalent to 0.1 parasites/ml of blood. All dilutions were treated with RNase inhibitor using RNase Out (Life Technologies) with a final concentration of 1 unit per µL. The same blood sample having 10^5^ parasites/ml was serially diluted as well in un-infected blood at intervals of ten-fold to reach a concentration of 10 parasites/ml of blood, then at intervals of two-fold dilution until reaching a concentration of 0.3 parasites/ml of blood. Infected blood (200 µL) was then mixed with 600 µL of DRS and RNA was extracted from all 800 µL as described above. RNA was eluted in a volume of 50 µL, of which 5 µL was used as a template in each qRT-PCR reaction.

### Statistical analysis

Demographic and survey items were used to assess differences in the likelihood of being an asymptomatic carrier of *P. falciparum* parasites. After stratification for demographic location by department, simple logistic regression models were used to determine the likelihood of carrying *P. falciparum* parasites using survey questions in Stata V.11 (Statacorp, College Station, TX, USA). Questions included items relating to participant knowledge of malaria, risk factors for malaria infection, demographic characteristics, and human mobility characteristics. Statistical significance was determined at an alpha level of 0.05 in all logistic regression models.

## Results

**Fig. 2:**
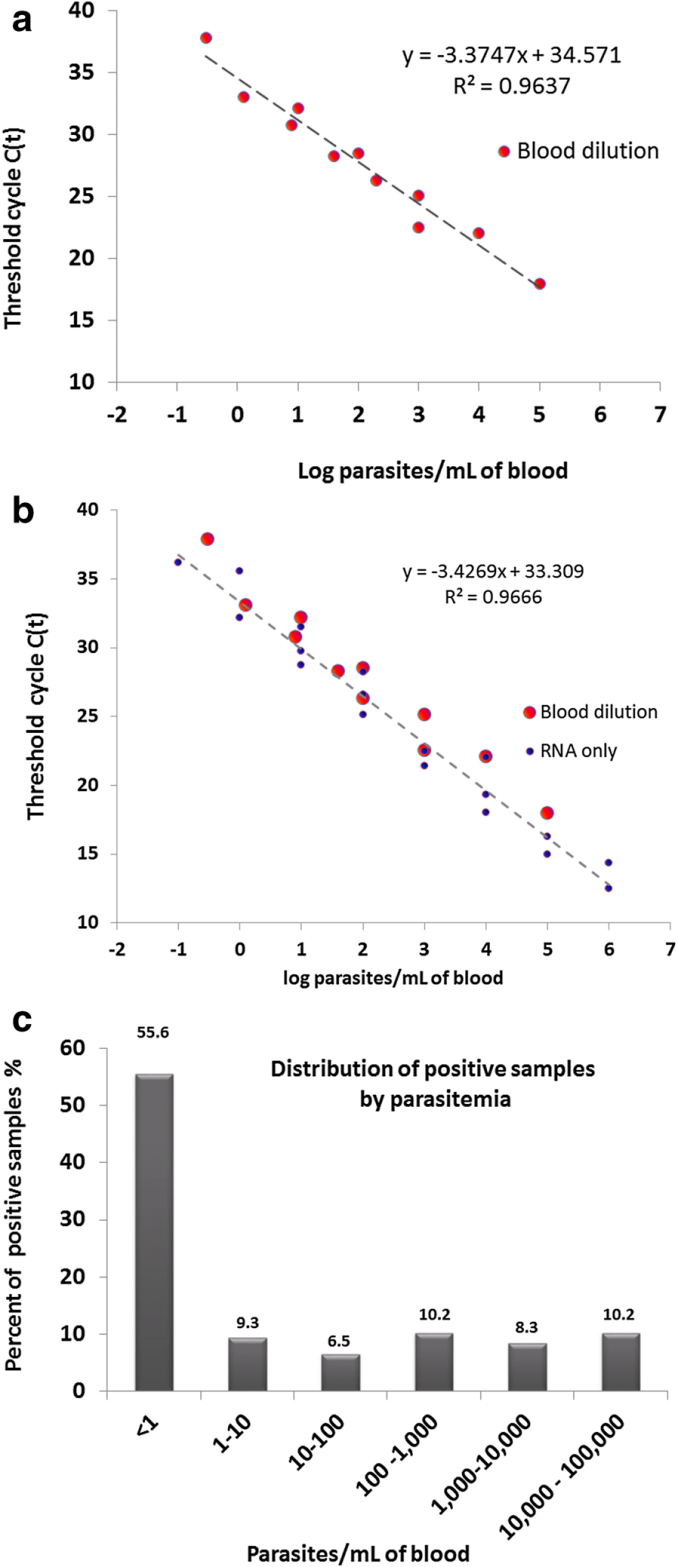
Estimating parasitaemia by qRT-PCR in field samples using standard curves generated from in vitro cultured ring stage parasites. **a** Standard curve by serial blood dilution; **b** standard curve combined serial dilution of blood and RNA; **c** distribution of positive samples by parasitaemia

Using the synchronized ring stage cultured parasites in a series of blood dilutions, the limit of detection of the qRT-PCR assay for *P. falciparum* was 0.0003 parasitized red cells per µL of whole blood (0.3 per mL) (Fig. 2a). In testing serial dilutions of RNA extracted from a blood sample with a higher parasitaemia, the results were highly correlated with RNA extracted from the blood dilution series, however the limit of detection for the diluted RNA was even lower, 0.0001 parasitized red cells per µL (0.1 per mL) (Fig. 2b). Assays to determine the limit of detection of this method were conducted three times independently with parasites from in vitro culture to demonstrate reproducibility before using it with field-collected samples. The standard curve obtained using RNA extracted from the blood dilution series (Fig. 2a) was used to estimate the parasitaemia of all field samples (Fig. 2c), since extraction of RNA from the blood samples used to prepare this curve most closely corresponds to the preparation of RNA from the field samples. Samples were collected from rural sites in six departments across Haiti, and all samples were tested for malaria parasites using RDT, microscopy and qRT-PCR. Among the 563 samples tested, ten and 16 were found positive for malaria by RDT and microscopy, respectively. Using the qRT-PCR test to assess the infection status of these subjects, an additional 92 were identified for a total of 108. The majority (55.6 %) of these infections hosted a parasitaemia of less than one parasite per ml of blood (Fig. 2c). Results by department for all tests are shown in Table 1. All samples testing positive by the qRT-PCR were re-extracted for total nucleic acid using a modified protocol of the same extraction kit as per the manufacturer’s protocol. The assay was then repeated using the same reaction conditions with and without the reverse transcription step to evaluate the difference in detecting rRNA and genomic DNA, respectively. By qPCR only 20 of the 108 qRT-PCR-positive samples were identified as positive.

**Table 1:** Performance of qRT-PCR compared to microscopy, and RDT with blood sample obtained from asymptomatic population

Location	RDT			Slide			RT-PCR		
Department	(n)	No. Pos.	% Pos.	(n)	No. Pos.	% Pos.	(n)	No. Pos.	% Pos.
Artibonite	108	0	0	108	0	0	108	3	2.8
Central Plateau	122	0	0	122	0	0	122	6	4.9
Grand Anse	194	10	5.1	194	14	7.1	194	79	40.7
Nord	39	0	0	39	1	2.6	39	10	25.6
Ouest	63	0	0	63	0	0	63	2	3.2
Sud-Est	37	1	2.7	37	2	5.4	37	8	21.6
*Total*	*563*	*14*	*2.4*	*563*	*17*	*3.0*	*563*	*108*	*19.1*

Overall 3.0 % of samples tested were positive for malaria by microscopic analysis, 2.4 % by RDT and 19.1 % by qRT-PCR. However, a large variation in prevalence of infection was detected across departments. Departments having a low prevalence of malaria infections (i.e., fewer than five sub-microscopic carriers per 100 in the population) are Artibonite, Central Plateau and Ouest. High prevalence (i.e., more than 20 sub-microscopic carriers per 100 in the tested population) was observed in the Nord, Sud-Est and Grand-Anse Departments. Thus, a large reservoir of asymptomatic, sub-microscopic carriers is present in various locations across the peninsula interspersed with departments with very low prevalence.

No significant risk factors emerged from the regression analysis for insecticide usage nor did sleeping outdoors during morning or night correlate with a positive qRT-PCR result. However, travelling outside the screening area within the last month was shown to be a protective factor with odds ratio of 0.53 and (P = 0.004). Those who were qRT-PCR-positive were more likely to know about malaria as a disease and its mode of transmission, with an odds ratio of 1.79 (P = 0.009). The odds of being positive for malaria by qRT-PCR when a household member was also infected were 2.2 times higher than for those who did not live near a malaria-infected family member, and this relationship was found to be statistically significant with a (P = 0.002). Lastly, the odds of being positive by qRT-PCR with a history of previous infection was 2.1 times higher than for those who were self-identified as not previously having been infected (P = 0.003). A summary of results for the demographic questionnaire is presented in Table 2.

**Table 2:** Logistic analysis of demographic factors for malaria parasite transmission

Exposure factors	Survey response	n	Proportion	Likelihood of positive qRT-PCR result			
				OR	P value	95% CI	OR
Bednet use	No	482	35.1	Ref	–	–	–
	Yes		64.9	1.49	0.099	0.927	2.409
Insecticide use	No	435	78.2	Ref	–	–	–
	Yes		21.8	0.93	0.803	0.528	1.639
Sleeping outside	No	450	62	Ref	–	–	–
	Yes		38	1.35	0.192	0.861	2.116
Has window nets	No	462	90.7	Ref	–	–	–
	Yes		9.3	1.08	0.845	0.512	2.267
Travel history	No	510	46	Ref	–	–	–
	Yes		54	0.53	0.004	0.345	0.817
Knowledge of malaria	No	490	61.2	Ref	–	–	–
	Yes		38.8	1.79	0.009	1.154	2.782
History of infection	No	502	79.7	Ref	–	–	–
	Yes		20.3	2.12	0.003	1.297	3.460
Household member infected	No	355	70.1	Ref	–	–	–
	Yes		29.9	2.21	0.002	1.339	3.633
History of treatment	No	387	73.9	Ref	–	–	–
	Yes		26.1	2.13	0.003	1.284	3.538

## Discussion

A survey method capable of identifying virtually every individual infected with the parasite will be crucial in the malaria elimination phase. It will allow making a reliable estimate of the proportion of sub-microscopic infections which must be factored in when developing intervention plans (e.g., mass drug administration or test and treat), and, secondly to test the efficacy of intervention measures after implementation to evaluate success [22]. PCR has been extensively used as the most sensitive survey tool with a detection limit of 0.01–0.2 parasites/µL of blood [20]. PCR depends on detecting genomic DNA, commonly a segment of the small sub-unit ribosomal RNA gene due to its multiple copy numbers and availability of relatively conserved regions. The power of detecting the ribosomal RNA itself has been recognized by others [22, 28–32], and by qRT-PCR the limit of detection is as low as 0.0003 parasites/µL of blood, as reported here. The limited use of qRT-PCR rather than conventional one step or nested PCR can be attributed to factors such as the potential challenge of maintaining RNA quality and the increased cost of using a probe-based assay. The value of the extremely low limit of detection offered by the qRT-PCR test employed here is seen in the large number of asymptomatic carriers identified which were undetected by any other method, including qPCR.

The qRT-PCR assay described here has the potential to detect blood stage infections from the first emergence of the parasite into the blood from the liver throughout the course of blood stage infection, even during what appears to be an asymptomatic, chronic phase. A single exo-erythrocytic (EE) stage schizont releases merosomes into the blood stream, which ultimately rupture releasing tens of thousands of merozoites. Assuming an average blood volume of 5 L and a single exoerythrocytic stage schizont releasing merozoites, then two or more infected red cells may be expected per ml of blood to initiate the asexual growth phase. To detect this parasitaemia, the test must have a limit of detection equal to or lower than 0.004 parasites/µL of blood. The qRT-PCR targeting the small ribosomal RNA of *P. falciparum* with a limit of detection of 0.0003 parasites/µL of blood exceeds this threshold by an order of magnitude. Comparing the qRT-PCR assay to qPCR on total nucleic acids using the same primer sets and reaction conditions, excluding the reverse transcription step, C_t_ values for qRT-PCR were an average of 14 lower than for qPCR for the same sample. The large number of rRNA molecules in each parasite (>10^4^) [33], their presence in free merozoites, and the fact that they are packaged separately in ribosomes which may be released as separate particles upon cell lysis, all likely contribute to the ability to detect as few as three parasitized red blood cells per 10 ml of blood, even when sampling only 0.4 ml of blood and ultimately assaying RNA from only 5 % of the blood sample.

In order for Haiti to reach the pre-elimination phase of malaria, the number of active cases must be less than one per 1000 population at risk in preparation for a reduction to zero locally acquired cases. Haiti would have to maintain zero locally acquired cases for at least 3 years prior to becoming certified malaria-free country by WHO [34]. The current status seems ambiguous, since official records indicate a reduced overall number of clinical cases from ~80,000 in 2010 to ~17,000 in 2014 [6] and hyperendemic foci of malaria remain despite current control efforts [7, 18]. All official published data acquired from hospitals and dispensaries across Haiti represent only data from febrile cases, yet in an area experiencing high transmission only 17 % of all acute febrile illnesses were attributed to malaria [7].

Comparatively high malaria prevalence has been reported previously in the Grande Anse, Nord and Sud-Est Departments [7, 18], however, none of the reported data accurately captured the sub-microscopic levels of infection. There is no national policy on active cross-sectional surveillance to monitor number of asymptomatic or sub-microscopic carriers of parasite in the country. Needless to say Haiti currently has no tools available to identify the sub-microscopic population, but counting the sub-microscopic infections found in these departments raises the already high prevalence to a remarkably high level. Over the past few years there has been an elevated level of malaria transmission in Grand-Anse. A community-based survey there found that gametocytes were rare in the asymptomatic population (0.9 %) [7], however the survey methods used to detect gametocytes have the same higher limit of detection as microscopy for malaria diagnosis as described here. Thus, it is expected that a higher percentage of gametocytaemic individuals would have been found in this population, if assessed by PCR-based methods. In the present study 40 % of the asymptomatic population in the same area were infected with the parasite. In spite of the low levels of parasitaemia in this population, it is likely over time that they produce gametocytes in sufficient quantities to contribute in disease transmission [20]. Given the large percentage of individuals having such a low parasitaemia (Fig. 2c), it is highly likely that they have chronic infections, with only a small fraction sampled at the very onset of a new malaria infection. Despite this logic, whether the parasites were persistently present in the blood stream from a previous infection or recurrent re-infections is yet to be studied. Further, the methodology used does not necessarily detect only viable parasites, rather stable nucleic acids derived therefrom, thus further analysis is required to be able to accurately estimate the parasite burden in individuals positive by the qRT-PCR test. The study is also limited having screened only females of child-bearing age. Whether or not the same level of high prevalence is seen in males, elders and children in the population requires further investigation.

In spite of the availability of bed nets to 64 % of the population, no statistical significance for their proposed role in protecting individuals from infection was found. This finding is consistent with the fact that the main vector for malaria transmission in Haiti is *An. albimanus,* an outdoor-biting mosquito with a preference for blood meals at dawn and dusk [35]. The survey showed that those with history of previous infection or with a household member that is or was infected are twice as likely to be positive for sub-microscopic infection. The results were statistically significant and come in agreement with the observation, that those who are closely associated with others infected with malaria are more likely to be infected. Finally, travel outside the screening site was a protective factor against infection. Further information relative to the pattern of travel, the length of absence, and travel destination(s) is needed to understand more clearly the basis of the protective effect, however it is consistent with the interpretation that transmission is focal within sites and that travel to other locations in Haiti where transmission is lower is protective.

There is a large variation in malaria prevalence among study sites, and these results suggest that using this highly sensitive qRT-PCR based survey tool would facilitate accurately mapping the reservoirs of malaria across the country. Given that samples obtained in a field setting are stable for transportation to established laboratories for rapid completion of the assay, the results can be obtained in a timely fashion for mapping purposes and for assessing effectiveness of elimination efforts. Obtaining such data will be crucial to effectively guiding the utilization of resources available for elimination and evaluating success.

## Conclusions

The findings of this study demonstrate the superior efficacy of the qRT-PCR as a surveillance tool in detecting malaria infections, including submicroscopic infections, as compared to other available tools frequently employed including PCR. DRS provides a highly useful preservative for parasite nucleic acids in field conditions, making this assay feasible for use under field conditions in underdeveloped countries by allowing successful transportation of stabilized samples to established laboratories for analysis. The study identified a large number of sub-microscopic infections in Haiti that were not previously described. Plans for malaria elimination on Hispaniola must account for the large number of asymptomatic, sub-microscopic infections present in Haitians in regions where malaria transmission is found.

## Abbreviations

CDC: Centers for Disease Control and Prevention
DNA: deoxyribonucleic acid
DRS: DNA/RNA Shield™
HRP2: histidine-rich protein 2
LNSP: Laboratoire National de Santé Publique
MSPP: Ministère de la Santé Publique et de la Population
PCR: polymerase chain reaction
qPCR: quantitative polymerase chain reaction
qRT-PCR: quantitative reverse transcription polymerase chain reaction
RDT: rapid diagnostic test
RNA: ribonucleic acid
